# Assessment of effect modification of statins on new-onset diabetes based on various medical backgrounds: a retrospective cohort study

**DOI:** 10.1186/s40360-019-0314-x

**Published:** 2019-05-28

**Authors:** Keiko Yamazaki, Yasuo Takahashi, Kotoe Teduka, Tomohiro Nakayama, Yayoi Nishida, Satoshi Asai

**Affiliations:** 1 Division of Genomic Epidemiology and Clinical Trials, Clinical Trials Research Center, Nihon University School of Medicine, 30-1 Oyaguchi-Kami Machi, Itabashi-ku, Tokyo, 173-8610 Japan; 2 Division of Laboratory Medicine, Department of Pathology and Microbiology, Nihon University School of Medicine, 30-1 Oyaguchi-Kami Machi, Itabashi-ku, Tokyo, 173-8610 Japan; 3 Division of Pharmacology, Department of Biomedical Sciences, Nihon University School of Medicine, 30-1 Oyaguchi-Kami Machi, Itabashi-ku, Tokyo, 173-8610 Japan

**Keywords:** Retrospective cohort study, Clinical database, New-onset diabetes mellitus, Statin, Propensity-score matching

## Abstract

**Background:**

The aim of this study was to investigate the association between statin use and new-onset diabetes in clinical settings and to assess its effect modification (heterogeneity) among patients with various medical histories and current medications.

**Methods:**

In a total of 12,177 Japanese patients without diabetes, from December 2004 to November 2012, we identified 500 statin users and 500 matched non-users using propensity-score matching. Patients were followed until December 2017. We estimated the hazard ratios of new-onset diabetes associated with statin use. We also tested the heterogeneity of the treatment effect by evaluating subgroup interactions in subgroups according to sex, age, medical history, and current medication.

**Results:**

New-onset diabetes had occurred in 71 patients (13.6%) with statin use and 43 patients (8.3%) with non-use at 5 years (hazard ratio, 1.66; 95% confidence interval [CI], 1.11 to 2.48; *P* = 0.0143), and in 78 patients (15.6%) with statin use and 48 patients (9.6%) with non-use at 10 years (hazard ratio, 1.61; 95% CI, 1.10 to 2.37; *P* = 0.0141). There were no significant treatment-by-subgroup interactions in all subgroups defined according to sex, age, medical history, and current medication.

**Conclusions:**

In patients with various clinical backgrounds, those who received statin therapy had a higher risk of new-onset diabetes at 5 and 10 years than those who did not receive it. Effect modification of statins on new-onset diabetes was not found in patient populations defined according to various comorbid diseases or concomitant drugs.

## Background

The use of 3-hydroxy-3-methylglutaryl coenzyme A (HMG-CoA) reductase inhibitors, known as statins, can effectively reduce cardiovascular events and mortality [1, 2]. Current guidelines, such as the National Institute for Health and Clinical Excellence (NICE) guidelines [3] and the 2013 American College of Cardiology/American Heart Association (ACC/AHA) guidelines [4], recommend statins for primary and secondary prevention of cardiovascular disease as assessed with a recommended risk score. Although statins are generally considered to be safe and well tolerated [5], there is concern about the relation between the use of statins and the development of diabetes mellitus [6–9]. Randomized controlled trials and meta-analyses have reported unfavorable results that statin therapy is associated with an increased incidence of new-onset diabetes [10–13]. The effect of statins on the development of diabetes appears to be dose-dependent and differentiated between different types of statins [14–18], and to be associated with adherence and duration of therapy [15, 19]. Recent observational studies reported that increased incidence of new-onset diabetes with statin use has also been seen in particular patient populations, including women, healthy adults, and East Asian people [20–24].

In clinical practice, all complications and comorbid conditions, i.e., the clinical characteristics, as well as cardiovascular risk factors, should be assessed before starting statin therapy, although comorbid conditions, including hypertension, obesity, and diabetes mellitus, which are commonly observed in patients with dyslipidemia, are major risk factors for cardiovascular disease. Therefore, whether the effect of statins on glycemic control may vary in particular patient populations defined according to various comorbid diseases or concomitant drugs, such as cardiovascular disease, hypertension and medications for these conditions, would be of interest. There is, however, a paucity of reports providing data from a comprehensive analysis of medical history and current medication, which may modify the effect of statins on new-onset diabetes.

The aim of the present study was to examine whether statin therapy could increase the risk of new-onset diabetes among patients with various backgrounds in clinical settings and to assess its effect modification (heterogeneity) in various subgroups defined by sex, age, medical history, and current medication, using a clinical database in Japan.

## Methods

### Data source

We obtained the study data from December 1, 2004 to December 31, 2017 using the Nihon University School of Medicine (NUSM) Clinical Data Warehouse (CDW), which is a centralized data repository that integrates separate databases, including an order entry database and a laboratory results database, from the electronic medical record system at three hospitals affiliated with NUSM, and is described elsewhere [25]. The prescription database in the CDW contains information from approximately 0.8 million patients, and prescribing data are linked longitudinally to detailed clinical information such as patient demographics, diagnosis, and laboratory data. To protect patient privacy, patient identifiers are replaced with anonymous identifiers in all databases of this CDW. Several epidemiological studies in various therapeutic areas using NUSM’s CDW have been published [26–33].

The experimental protocol was approved by the Ethical Committee of Nihon University School of Medicine, and the study was conducted in compliance with the Helsinki Declaration and the Ethical Guidelines for Medical and Health Research Involving Human Subjects of the Ministry of Education, Culture, Sports, Science and Technology and the Ministry of Health, Labour and Welfare, Japan. No informed consent was required because this was a retrospective observational study using anonymized archived data from a clinical database and did not compromise anonymity or confidentiality.

### Study design and population

This was a retrospective cohort study evaluating the effects of statin versus no statin treatment on new-onset diabetes in patients with different medical histories. The study was divided into two periods: 1) an entry period (December 1, 2004 to November 30, 2012), which was used for selection of study subjects and description of baseline characteristics; and 2) a follow-up period (from the index date as defined below until December 31, 2017), which was used to capture outcome events.

The cohorts identified for the study included Japanese patients at Nihon University Itabashi Hospital aged 30 to 85 years, and who met the following criteria:

1. At least one outpatient visit to undergo laboratory tests, including plasma glucose or hemoglobin A1c (HbA1c), during both the entry and follow-up periods

We identified treatment groups who fulfilled the following criteria:

1. Statin users: patients who had been newly treated with a statin for at least 90 days during the entry period as described previously [22, 24]. The index date was defined as the date of the first prescription of a statin. We excluded patients who received a statin for less than 90 days or who had been newly treated with a statin after December 1, 2012 (outside the entry period).
2. Statin non-users: patients who did not receive a statin during the study period (entry and follow-up periods), and were followed up for at least 90 days after the index date, which was defined as the earliest date of a blood test for either plasma glucose or HbA1c during the entry period

We excluded patients who met the following criteria:

1. Patients who had schizophrenia or renal failure, or who had been treated with immunosuppressive drugs or steroids during the study period.
2. Patients with a diagnosis of diabetes or prescribed medication for diabetes prior to the index date.
3. Patients with missing values of serum triglyceride data during the 90 days preceding the index date.
4. Patients who fulfilled the following criteria for test results prior to the index date: either elevated casual plasma glucose level ≥ 126 mg/dl, or locally measured 2 h glucose ≥200 mg/dl following a 75 mg oral glucose tolerance test (OGTT) or elevated HbA1c ≥6.5%.

**Fig. 1:**
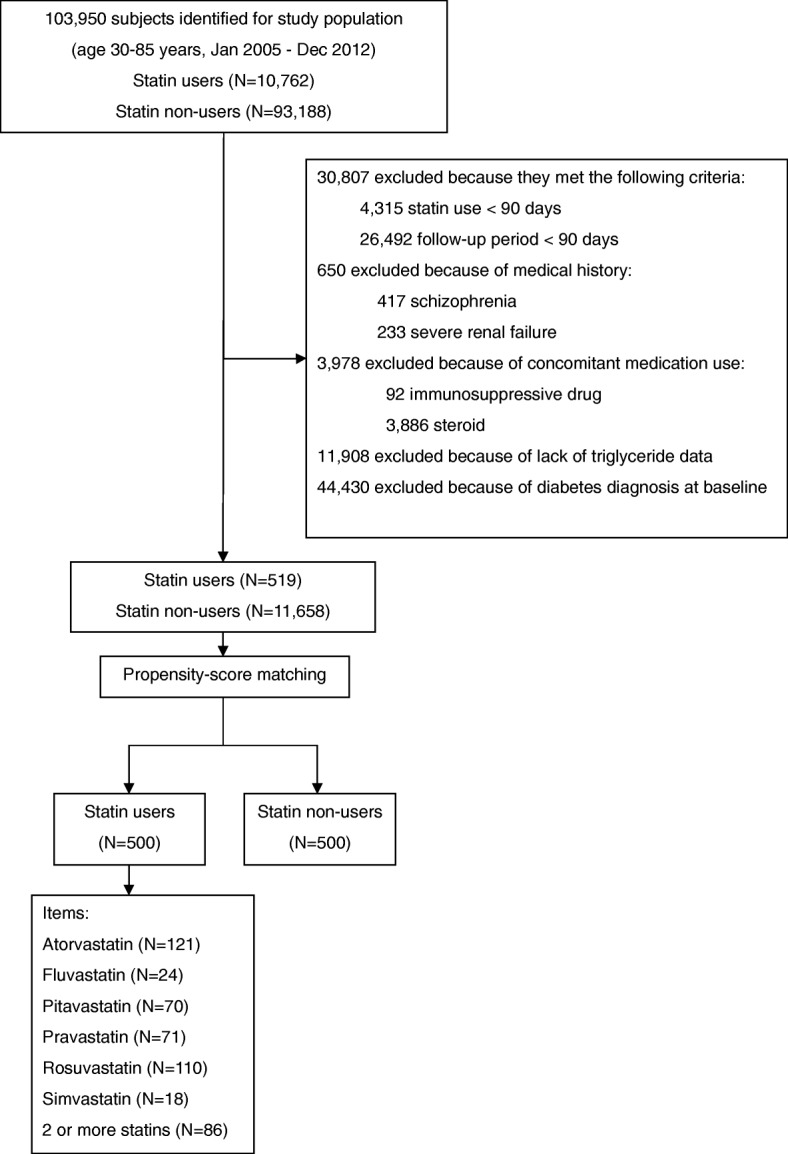
Identification of study population Cohorts of statin users and non-users were matched using propensity-score matching after a screening procedure (i.e., some patients were excluded for the reasons shown in the figure)

Consequently, we identified 519 new users of statins and 11,658 statin non-users who fulfilled the above criteria (Fig. 1). Then, we identified an equal number of statin users (*N* = 500) and matched non-users (*N* = 500) after propensity-score matching, and compared them.

### Outcome

We defined our diabetes endpoints as follows:

1. Clinical diagnosis of diabetes in combination with at least one blood test result as follows: either elevated casual plasma glucose level ≥ 200 mg/dl or locally measured 2 h glucose ≥200 mg/dl following a 75 mg OGTT or elevated HbA1c ≥6.5%, according to the Committee for the Classification and Diagnosis of Diabetes Mellitus of the Japan Diabetes Society [34].
2. Initiation of insulin or an oral hypoglycemic drug

Patients were followed from 91 days after the index date until the diabetes endpoint occurred, or were assessed up to the final visit (censored). We created two datasets of 5-year and 10-year follow-up data to perform long-term analysis at different time points.

### Covariates

For each individual, information on patient demographics (age and sex), medical history, current medication, and laboratory results was collected. Medical history included information on cerebrovascular disease (ICD-10 codes, I60-I69), ischemic heart disease (I20-I25), other heart disease (I30-I52), liver disease (K70-K77), kidney disease (N00-N19), rheumatoid arthritis (M5, M6), and hypertension (I10) that had been diagnosed prior to the index date. We recorded current users of medication including antihypertensive agents (including angiotensin receptor blockers (ARB), angiotensin converting enzyme (ACE) inhibitors, β-blockers, calcium channel blockers (CCB), antihypertensive diuretics and other antihypertensive drugs), lipid-lowering drugs (including fibrates, bile acid sequestrants, nicotinic acid, cholesterol absorption inhibitors and other lipid-lowering drugs), antithrombotic drugs, liver disease therapeutics, kidney disease therapeutics, non-steroidal anti-inflammatory drugs (NSAID), proton pump inhibitors (PPI), histamine2-receptor antagonists (H2 blockers), non-thiazide diuretics and anti-arrhythmic drugs, defined as patients who had received these agents in the 90 days preceding the index date.

Also, blood test data (triglyceride and casual plasma glucose) were collected for each individual during the 90 days preceding the index date.

### Propensity-score matching

Because this study was an observational study, which involves inherent issues of selection bias, we used propensity score matching (greedy 1:1 matching) to reduce bias by balancing covariates between statin users and non-users. This method is an effective tool to reduce bias in nonrandomized studies [35, 36], and is described elsewhere [37]. In brief, the propensity score for each subject was obtained by fitting a logistic regression model that included the predictor variable as an outcome and baseline covariates including follow-up period, age, sex, medical history, current medication, and baseline levels of triglyceride and casual glucose, as shown in Table 1. After the propensity score was constructed, we matched the propensity score of each group of statin users and non-users. A nearest-neighbor-matching algorithm with a “greedy” heuristic was used to match patients with a caliper of 0.2 standard deviations of the logit of the propensity score.

**Table 1:** Baseline characteristics of study population after propensity-score matching

Characteristics	Statin users	Statin non-users	*P* value
	(*n* = 500)	(*n* = 500)	
Age (years, mean ± sd)	60.0 ± 10.9	61.2 ± 13.8	0.1241
Women	281 (56.2)	289 (57.8)	0.6094
Medical history			
Cerebrovascular disease	75 (15.0)	76 (15.2)	0.9296
Ischemic heart disease	115 (23.0)	102 (20.4)	0.3186
Other heart disease	112 (22.4)	101 (20.2)	0.3955
Rheumatoid arthritis	15 (3.0)	18 (3.6)	0.5954
Liver disease	68 (13.6)	68 (13.6)	1.0000
Kidney disease	20 (4.0)	25 (5.0)	0.4456
Hypertension	103 (20.6)	82 (16.4)	0.0872
Medication			
Antihypertensive drugs	216 (43.2)	194 (38.8)	0.1572
ARB	110 (22.0)	112 (22.4)	0.8790
ACEI	19 (3.8)	20 (4.0)	0.8702
Beta blocker	35 (7.0)	33 (6.6)	0.8016
CCB	124 (24.8)	120 (24.0)	0.7684
Antihypertensive diuretic	4 (0.8)	2 (0.4)	0.4128
Other antihypertensive drugs	57 (11.4)	57 (11.4)	1.0000
Lipid-lowering drugs other than statins			
Fibrate	13 (2.6)	15 (3.0)	0.7014
Bile acid sequestrant	4 (0.8)	4 (0.8)	1.0000
Nicotinic acid	7 (1.4)	5 (1.0)	0.5613
Cholesterol absorption inhibitor	1 (0.2)	0 (0.0)	0.3171
Other lipid-lowering drugs	16 (3.2)	13 (2.6)	0.5718
Antithrombotic drug	182 (36.4)	193 (38.6)	0.4724
Liver disease therapeutic	11 (2.2)	9 (1.8)	0.6514
Kidney disease therapeutic	4 (0.8)	6 (1.2)	0.5250
Proton pump inhibitor	92 (18.4)	79 (15.8)	0.2749
H2 blocker	60 (12.0)	70 (14.0)	0.3471
NSAID	129 (25.8)	131 (26.2)	0.8854
Non-thiazide diuretic	35 (7.0)	34 (6.8)	0.9007
Antiarrhythmic drug	45 (9.0)	46 (9.2)	0.9124
Laboratory parameters			
Triglyceride (mg/dL, mean ± sd)	134.7 ± 65.4	134.4 ± 75.8	0.9494
Casual glucose (mg/dL, mean ± sd)	102.2 ± 10.0	101.8 ± 10.6	0.6250

Data are numbers of individuals (%) unless otherwise stated. Comparisons of differences in patient characteristics between groups were performed using t-test for continuous variables and chi-squared test for categorical data. Abbreviations: *ARB* Angiotensin type II receptor blocker, *CCB* Calcium channel blocker, *ACEI* Angiotensin-converting enzyme inhibitor, *H2* Blocker, histamine2-receptor antagonist, *NSAID* Non-steroidal anti-inflammatory drug

### Statistical analysis

After propensity-score matching, we used *t*-test for continuous variables and chi-squared test for categorical data to compare differences in baseline characteristics between statin users and non-users. Diabetes-free survival curves were constructed by means of Kaplan–Meier methods, and differences between the treatment groups were evaluated using the log-rank test. Cox proportional-hazard regression was used to estimate the hazard ratios and 95% confidence intervals (CI) of new-onset diabetes associated with statin use. Also, Cox regression models were used to evaluate the effect of statins on new-onset diabetes in subgroups defined according to sex (male or female), age groups (< 65 or ≥ 65 years), medical history (presence or absence), and current medication (use or non-use). In addition, we tested the heterogeneity of the treatment effect by evaluating treatment-by-subgroup interactions in the Cox model. Hazard ratio in all analyses was adjusted for age and baseline levels of triglyceride and casual glucose. All reported *P* values are two-sided, and an alpha level of 0.05 was considered to indicate statistical significance. All statistical analyses were performed with SAS software, version 9.3 (SAS Institute Inc., Cary, NC).

## Results

### Study subjects

Based on our initial inclusion and exclusion criteria, we identified a total of 12,177 patients for this study; 519 statin users and 11,658 non-users. After propensity-score matching, the study included 500 statin users and 500 matched non-users (Fig. 1). The mean follow-up in all subjects was 150.4 weeks; the length (mean ± standard error) of follow-up was likely to be longer in statin non-users (152.6 ± 6.4 weeks) than in statin users (148.3 ± 6.4 weeks), but the difference between them was not significant. During the follow-up period, 121 patients were exposed to atorvastatin, 24 to fluvastatin, 70 to pitavastatin, 71 to pravastatin, 110 to rosuvastatin, 18 to simvastatin, and 86 to two or more types of statins. Table 1 shows the baseline characteristics of the patients after propensity-score matching. In statin users, mean age was 60.0 years and 56.2% were women. Statin non-users were older, but showed a similar proportion of women to statin users; mean age was 61.2 years and 57.8% were women. There were no significant differences in medical history and current medication between statin users and non-users. Approximately half of each cohort had a history of hypertension, and one-fifth had a history of ischemic heart disease or other heart disease. More than two-fifths of each cohort took an antihypertensive drug, approximately one-third took an antithrombotic drug, and approximately one-fourth took an NSAID. In laboratory parameters, there was no significant difference in triglyceride and casual glucose levels between statin users and non-users.

### Risk of new-onset diabetes

**Fig. 2:**
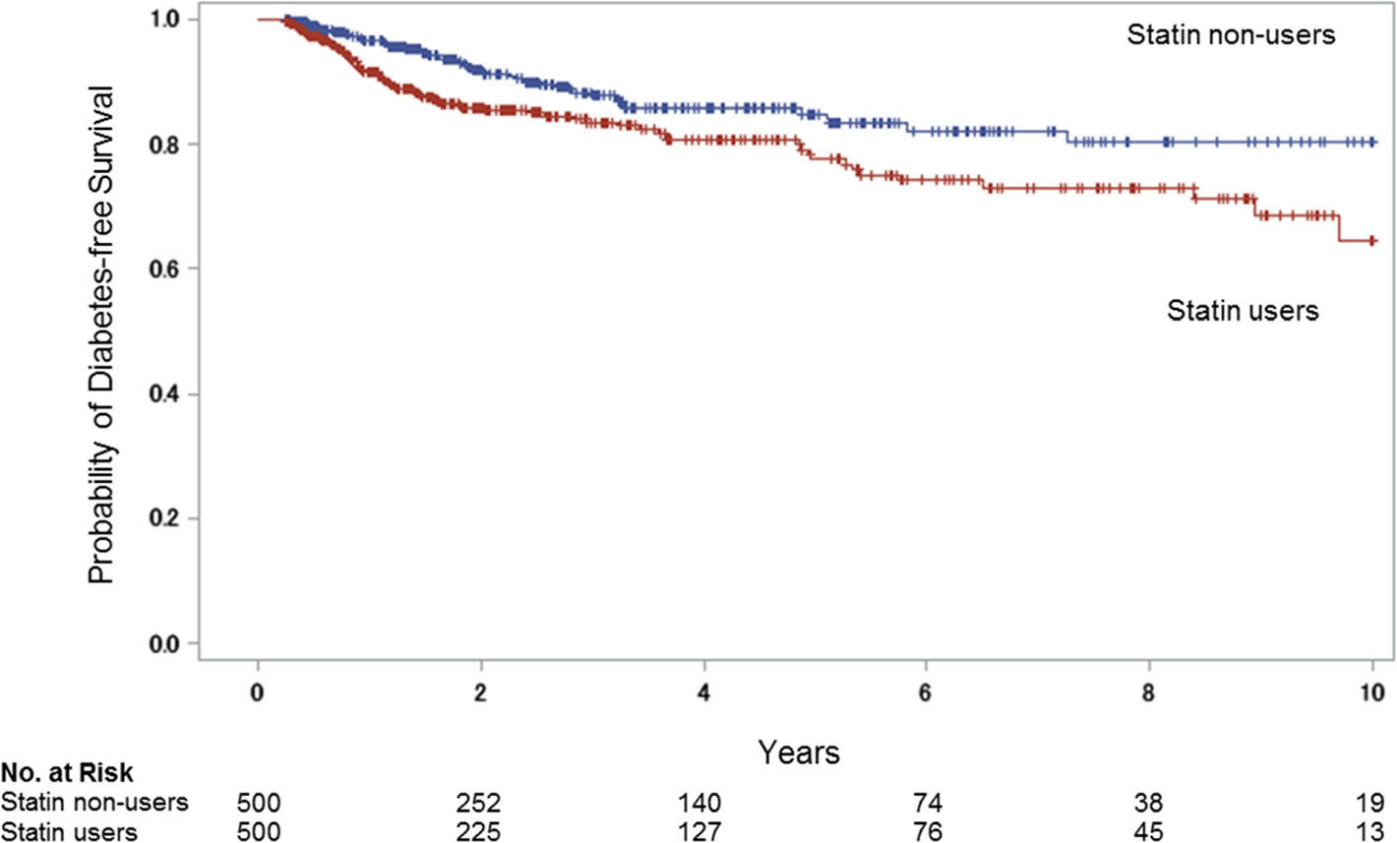
Kaplan–Meier plot of new-onset diabetes-free survival in statin users and non-users Kaplan–Meier survival curves showed a significantly higher occurrence rate of new-onset diabetes in the statin user group than in the matched non-user group (*P*  <  0.001, log-rank test). Tick marks indicate censored data

New-onset diabetes had occurred in 71 patients (13.6%) with statin use and 43 patients (8.3%) with non-use at 5 years (adjusted hazard ratio, 1.66; 95% confidence interval [CI], 1.12 to 2.48; *P* = 0.0143) (Table 2). At 10 years, new-onset diabetes had occurred in 78 patients (15.6%) with statin use and 48 patients (9.6%) with non-use (hazard ratio, 1.61; 95% CI, 1.10 to 2.37; *P* = 0.0141). Figure 2 shows the Kaplan–Meier plot for new-onset diabetes-free survival in statin users and non-users. Kaplan–Meier survival curves showed a higher occurrence rate of the endpoint of new-onset diabetes in statin users (*P*  <  0.001, log-rank test). Table 3 shows the hazard ratio for new-onset diabetes at 10-year follow-up, according to subgroups. There were no significant treatment-by-subgroup interactions in subgroups defined according to sex, age group, medical history, and current medication, although the number of patients with available data for analysis limited the power to determine interactions.

**Table 2:** Hazard ratio for new-onset diabetes for statin users versus non-users

Outcome	Statin users (*N* = 500)	Statin non-users (*N* = 500)	Unadjusted		Adjusted^a^	
	*No. of events (%)*		HR (95% CI)	*P* value	HR (95% CI)	*P* value
New-onset diabetes at 5 years	71 (13.6)	43 (8.3)	1.74 (1.20–2.55)	0.0039	1.66 (1.11–2.48)	0.0143
New-onset diabetes at 10 years	78 (15.6)	48 (9.6)	1.69 (1.19–2.44)	0.0040	1.61 (1.10–2.37)	0.0141

^a^Hazard ratios (HR) and 95% confidence intervals (CI) were estimated using Cox hazards models adjusted for age, and baseline levels of triglyceride and casual blood glucose

**Table 3:** Hazard ratio for new-onset diabetes, according to subgroup

Subgroup		No. of patients	Statin users	Non-users	Hazard ratio	*P* value for interaction
			*No. of events (%)*		(95% CI)	
All patients		1000	78 (15.6)	48 (9.6)	1.61 (1.10–2.37)	
Age						
< 65 yr		596	46 (14.1)	22 (8.2)	1.52 (0.90–2.58)	0.8219
≥ 65 yr		404	32 (18.4)	26 (11.3)	1.67 (0.93–2.99)	
Sex						
Female		581	39 (13.7)	25 (8.4)	1.66 (0.94–2.92)	0.9035
Male		431	38 (17.1)	22 (10.5)	1.58 (0.94–2.66)	
Medical history						
Cerebrovascular disease						
Yes		151	13 (17.3)	9 (11.8)	1.00 (0.37–2.66)	0.2935
No		849	65 (15.3)	39 (9.2)	1.77 (1.17–2.70)	
Ischemic heart disease						
Yes		217	29 (25.2)	12 (11.8)	2.28 (1.11–4.66)	0.2279
No		783	49 (12.7)	36 (9.1)	1.34 (0.84–2.14)	
Other heart disease						
Yes		213	25 (22.3)	12 (11.9)	1.59 (0.78–3.24)	0.9372
No		787	53 (13.7)	36 (9.0)	1.54 (0.97–2.44)	
Liver disease						
Yes		136	4 (5.9)	6 (8.8)	1.45 (0.65–3.27)	0.7781
No		864	74 (9.7)	42 (9.7)	1.65 (1.10–2.44)	
Hypertension						
Yes		450	44 (18.9)	26 (12.0)	1.57 (0.87–2.81)	0.9993
No		550	34 (12.7)	22 (7.8)	1.57 (0.87–2.64)	
Medication						
ARB use						
Yes		410	39 (18.1)	22 (11.3)	1.31 (0.78–2.21)	0.2802
No		590	39 (13.7)	26 (8.5)	1.87 (1.16–3.01)	
CCB use						
Yes		222	25 (22.7)	13 (11.6)	1.31 (0.70–2.46)	0.4316
No		778	53 (13.6)	35 (9.0)	1.72 (1.13–2.63)	
Beta blocker use						
Yes		244	23 (18.6)	12 (10.0)	1.61 (0.84–3.08)	0.9948
No		756	55 (14.6)	36 (9.5)	1.62 (1.06–2.47)	
Other antihypertensive drug use						
Yes		68	8 (22.9)	5 (15.2)	0.84 (0.33–2.12)	0.1378
No		932	70 (15.1)	43 (9.2)	1.71 (1.15–2.54)	
Antithrombotic drug use						
Yes		114	11 (19.3)	9 (15.8)	1.15 (0.41–3.20)	0.4685
No		886	67 (15.1)	39 (8.8)	1.73 (1.14–2.63)	
Proton pump inhibitor use						
Yes		375	36 (19.8)	26 (13.5)	1.40 (0.80–2.43)	0.5513
No		625	42 (13.2)	22 (7.2)	1.89 (1.09–3.27)	
H2 blocker use						
Yes		260	24 (18.6)	17 (13.0)	1.57 (0.77–3.21)	0.8787
No		740	54 (14.6)	31 (8.4)	1.68 (1.06–2.66)	
NSAID use						
Yes		171	26 (28.3)	10 (12.7)	2.95 (1.36–6.39)	0.0786
No		829	52 (12.8)	38 (9.0)	1.31 (0.83–2.06)	
Non-thiazide diuretic use						
Yes		135	14 (23.3)	9 (12.9)	1.38 (0.52–3.70)	0.7361
No		870	64 (14.5)	39 (9.1)	1.66 (1.10–2.51)	
Antiarrhythmic drug use						
Yes		69	6 (17.1)	7 (20.6)	0.67 (0.19–2.42)	0.1553
No		931	72 (15.4)	41 (8.8)	1.78 (1.19–2.68)	

Hazard ratios and 95% confidence intervals (CI) were estimated using Cox hazards models adjusted for age, sex, and baseline levels of triglyceride and casual glucose. *P* values for heterogeneity were obtained by fitting interaction terms. Data of subgroups whose hazard ratios could not be calculated because of small samples are not shown

Abbreviations: ARB, angiotensin type II receptor blocker; CCB, calcium channel blocker; H2 blocker, histamine2-receptor antagonist; NSAID, non-steroidal anti-inflammatory drug

## Discussion

In this study, we found that patients with various medical backgrounds who received statin therapy had a higher risk of new-onset diabetes at 5 and 10 years, compared with non-users. The hazard ratios of statin use for new-onset diabetes at 5 years and 10 years were similar, 1.66 and 1.61, respectively. However, effect modification (heterogeneity) of statins on new-onset diabetes was not found in various subgroups defined by stratification factors including sex, age, medical history, and current medication. These findings suggest that the effect of statins on the development of diabetes may manifest even in patients with various backgrounds, such as various comorbid diseases or concomitant drugs.

Much evidence from post hoc analyses from large clinical trials, meta-analyses, or observational studies confidently shows a consistent but weak association between statin therapy and the development of new-onset diabetes mellitus [6–9]. Although the precise links responsible for the increased risk of diabetes onset with statin therapy are still unknown, some mechanisms have been postulated. Statins have a suppressive effect on isoprenoid synthesis, resulting in decreased expression of glucose transport type (GLUT)-4, impairing glucose tolerance [38]. Moreover, statins suppress glucose-induced Ca^2+^ signaling pathways, leading to down-regulation of pancreatic beta-cell function and insulin secretion [39]. In this study, we identified a weak association between statin therapy and an increased risk of new-onset diabetes at 5 years (hazard ratio, 1.66; 95% CI, 1.12 to 2.48) and at 10 years (hazard ratio, 1.61; 95% CI, 1.10 to 2.37), in a Japanese cohort. As expected, these findings, which were consistent with previous reports, are reasonable. However, our estimates were likely to be higher than previous estimates from post hoc analyses from large clinical trials and meta-analyses [6–9]. The discrepancy between the present study and previous studies may be explained in part by differences in the experimental design or the study population (described in the following limitations paragraph). Our study was an observational study using non-randomized data and included patients with various backgrounds and clinical settings, who had comorbid conditions and were treated in our hospital. Therefore, the possibility that these would influence the results of this study cannot be ruled out. Furthermore, our finding that the hazard ratios of statin use for new-onset diabetes at 5 and 10 years were similar suggests a possible long-term effect of statin use on the development of new-onset diabetes mellitus.

Regarding the effect modification of statins on new-onset diabetes, we could not demonstrate any significant treatment-by-subgroup interaction in subgroups defined according to sex, age group, medical history, and current medication. There is a possibility that the statistical power may have been insufficient for assessing the interaction in some subgroups with a small sample size. However, this study showed that the hazard ratio of statin use for new-onset diabetes was higher in the subgroup with a history of ischemic heart disease than in the subgroup without, although the interaction between statin use and a history of ischemic heart disease was not significant. These results may suggest the potential increased risk of statin use for new-onset diabetes in patients with a history of ischemic heart disease. The underlying mechanism of these links between statin effect and history of ischemic heart disease responsible for the development of diabetes is not clear. The strongest predictors of new-onset diabetes include older age, higher blood glucose level, and features of the metabolic syndrome, such as obesity and raised triglycerides [6, 40]. These conditions are partially in common with risk factors for heart disorders, including coronary heart disease. Therefore, there is a possibility that statins may unmask diabetes, via which statins and these heart diseases themselves interact together, in people with a history of ischemic heart disease, who are more likely to develop diabetes. Our findings provide important clinical information to explain the diabetes risk in patients starting statin therapy, especially in those with a history of these heart diseases, although the benefit of statins to decrease cardiovascular risk completely outweighs the diabetes risk [18]. It is well-known that the reliability of subgroup analysis is likely to be reduced because of a combination of reduced statistical power and increased variance [40]. Therefore, the possibility that our findings of subgroup analysis may derive from the play of chance should be considered. Further studies with large samples will be needed to assess the effect modification of statin therapy on new-onset diabetes in patients with various backgrounds.

Our study has several limitations. It was a retrospective observational study with non-randomized data, which has some issues with respect to the potential for selection bias. We used rigorous statistical methods to balance potential confounding variables between statin users and non-users, including a propensity-score matching method. However, their ability to control for differences was limited to variables that were available or measurable. In this study, information on some biographical characteristics including smoking, alcohol consumption and family history of diabetes was not available, and we could not account for them. In addition, the model used in this study was not adjusted for body mass index (BMI) because of a large number of missing data of BMI in the study population. The possibility that this may have impacted on our results cannot be excluded, because individuals with higher BMI are more likely to develop new-onset diabetes [8]. However, BMI is well known to be closely related to serum triglyceride level. We, therefore, included baseline triglyceride level as a covariate for adjustment, minimizing the effect of unavailability of BMI. We also included age and baseline casual glucose level for adjustment, in addition to triglyceride level, because they are the strongest predictors of new-onset diabetes [6, 41]. Second, the dose of statins was not controlled and the type of statins was not assessed, because the population was small. However, the comparative effects of treatment with various statins, such as high potency vs low potency, lipophilic vs hydrophilic, or among individual statins, are of interest. When enough data are accumulated, further studies will be needed to determine the detailed effect of individual statins on new-onset of diabetes. Third, our study population included patients aged 30 to 85 years who attended our university hospital for various diseases, resulting in a higher prevalence of comorbidity in this study population than in the general population [42], potentially limiting the ability to generalize the results. Fourth, there was a possibility that we had underestimated the follow-up period in the statin group, because of the time-lag between the first prescription and blood test of HbA1c or glucose level. However, checks of laboratory parameters, including parameters of lipid metabolism, renal function, hepatic function, and glucose metabolism, are routinely performed when starting statin therapy in our hospital. Therefore, the impact of the difference between the first prescription date and the blood test date on the findings of our study may be trivial. The findings of our study call for further studies with large samples, such as longitudinal cohort studies for a long-term period and randomized clinical trials, for confirmation.

## Conclusions

In patients with various clinical backgrounds, in a real-world setting, those who received statin therapy had a higher risk of new-onset diabetes at 5 and 10 years than those who did not receive it. Effect modification of statins on new-onset diabetes was not found in patient populations defined according to various comorbid diseases or concomitant drugs.

## Data Availability

The datasets generated and analysed during the current study are not publicly available because approval was not obtained for the sharing of subject data from the Ethical Committee of NUSM. Data are however available from the corresponding author upon reasonable request and with permission of the Ethical Committee of NUSM.

## Abbreviations

ACE inhibitor: Angiotensin-converting enzyme inhibitor
ARB: Angiotensin II receptor blocker
CCB: Calcium channel blocker
CDW: Clinical Data Warehouse
CI: Confidence interval
H2 blocker: Histamine2-receptor antagonist
HbA1c: Hemoglobin A1c
HMG-CoA reductase inhibitor: 3-hydroxy-3-methylglutaryl coenzyme A reductase inhibitor
NSAID: Non-steroidal anti-inflammatory drug
NUSM: Nihon University School of Medicine
OGTT: Oral glucose tolerance test
PPI: Proton pump inhibitor
